# The King in the Crosshairs: Evidence of a Predation Attempt on European Bison by Wolves

**DOI:** 10.1002/ece3.73752

**Published:** 2026-05-29

**Authors:** Robin Rozemarijn Wijnands, Tomasz Borowik

**Affiliations:** ^1^ Mammal Research Institute Polish Academy of Sciences Białowieża Poland

**Keywords:** Białowieża primaeval Forest, *Bison bonasus*, *Canis lupus*, large carnivore, large herbivore, predator–prey interactions

## Abstract

The European bison ( *Bison bonasus* ), often referred to as ‘the king of the forest’, is a species with a unique and complex conservation story. The large mammal is often described as a non‐prey species, serving as a baseline reference in comparative ecological studies. However, historical data and our recent documented observation of wolves ( *Canis lupus* ) attacking a European bison herd suggest that they can be subjected to wolf predation. To our knowledge, we present the first video‐recorded evidence of wolves attacking a European bison herd in the Białowieża Primaeval Forest, focusing on a newborn calf. Although the video did not capture a direct kill, it suggests that other attacks on European bison could potentially be successful. This raises questions about what this could mean for conservation and management of both species and the extent of European bison‐wolf interactions. If wolf predation on bison occurs more frequently than presumed, it could potentially have ecological consequences.

## Graphical abstract

To our knowledge, we present the first video‐recorded evidence of wolves attacking a European bison herd in the BPF. The European bison, often referred to as the king of the forest, is generally described as a non‐prey species, but historical data and our recent documented observation provide clear evidence that they can be subjected to wolf predation. We discuss potential implications for conservation and management and potential ecological consequences if these predation events occur more frequently than presumed.

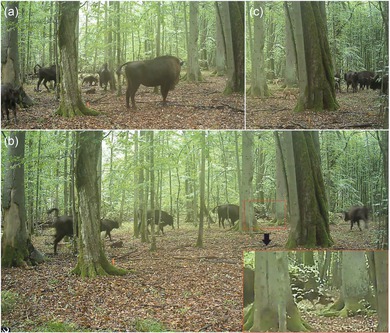

## 1 Introduction

Predation is a fundamental phenomenon shaping various ecological processes, from ecosystem to individual scales (Schmitz 2017). It influences population size and structure, impacts nutrient cycling, triggers physiological stress responses, and pushes evolutionary adaptations (Abrams 2000; Leroux and Schmitz 2015; Schmitz 2017; Xu et al. 2019). Beyond its lethal effects, predation also induces a wide range of non‐lethal responses in prey species, for instance through shifts in activity patterns, increased vigilance and changes in habitat selection (Lima and Bednekoff 1999; Laundré et al. 2001, 2014; Kuijper et al. 2013). However, the strength and nature of both lethal and non‐lethal effects depend on ecological context, such as environmental conditions, the composition of predator communities with different hunting strategies, and the traits of prey species involved (Gervasi et al. 2012; Kuijper et al. 2016; Bonnot et al. 2020).

Body size is another key determinant of predator–prey interactions, often constraining prey selection (Clements et al. 2014). According to the optimal foraging theory, predators should select for prey that offer the highest energetic benefits and the lowest energetic costs (MacArthur and Pianka 1966; Charnov 1976). In multi‐prey systems, this generally leads to a preference for smaller and easier species when they are available (Sinclair et al. 2003; Annear et al. 2023). Additionally, prey possessing morphological defences or behavioural strategies (e.g., living in groups), may further reduce their vulnerability to predation (Hayward and Kerley 2005). Nevertheless, even when preferred prey are available, opportunistic predation on non‐preferred prey can still occur (Barnardo et al. 2020). Particularly juveniles of non‐preferred prey could provide a valuable option, since they are small and vulnerable, presenting an opportunity for predators to extend prey selection (Annear et al. 2023).

The European bison provides an example of how prey status can shift over time. While historically preyed upon by brown bears ( *Ursus arctos* ) and wolves, in recent times it is often classified as a non‐prey species (Samojlik et al. 2018; Churski et al. 2021). The history of the European bison, often referred to as ‘the king of the forest’, in the Białowieża Primaeval Forest (BPF) has been relatively well documented and shows high population dynamics. From the 14th to the 20th century, the BPF was a favoured hunting ground for monarchs due to its abundance of game species, including the European bison (Krasińska and Krasiński 2013). During this entire described period, information about mortality and population numbers was well recorded, making the story of the European bison a special case in conservation history. Although wolves killed “only” eight bison on a yearly basis during the period 1840–1849, they were considered a threat to the management of bison and therefore were a target of persecution (Samojlik et al. 2018, 2019). However, after this period there were still reports of wolves killing bison, specifically calves (Samojlik et al. 2019). The European bison went extinct in the wild in 1919, and after its reintroduction in the BPF in 1952, the reporting of wolves killing or attacking bison decreased, with the first official record of a bison kill in the winter of 1994–1995 (Jędrzejewska and Jędrzejewski 2001).

In more recent times, it is often mentioned that the European bison generally has no predators, apart from humans (Jędrzejewska et al. 1997; Jędrzejewski et al. 2002), likely due to its large body size and the availability of easier prey for predators. With the number of bison kills by wolves being negligible compared to other prey species (Jȩdrzejewski et al. 2000), the bison is often labelled as a non‐prey species and used as a baseline species in comparative ecological studies (e.g., Churski et al. 2021). The lethal effects of predators on European bison appear to be very limited. Since predation risk differs in space and time, prey live in a ‘landscape of fear’, where certain moments in time and space are considered risky, and therefore avoided by prey (Laundré et al. 2001; Kuijper et al. 2013). So far, there is no evidence that a landscape of fear induced by natural predators affects bison behaviour and space use in the BPF (Theuerkauf and Rouys 2008). This could be due to the fact that wolf diet in the BPF mostly consisted of roe deer ( *Capreolus capreolus* ), red deer ( *Cervus elaphus* ) and wild boar ( *Sus scrofa* ) (Jędrzejewski et al. 2002). Only a small portion of wolf diet involved European bison, and this occurred through scavenging on bison carcasses, with bison accounting for one‐fourth of all scavenging cases. Only one kill was documented in the timespan of 10 years (Jȩdrzejewski et al. 2000), indicating that bison kills by wolves are rare.

Nonetheless, on the North American continent, the American bison ( *Bison bison* ) has been described as a prey species for wolves (Smith et al. 2000; Jung 2011). Although these large mammals are not the wolves' main prey, likely because predating bison carries substantial risk, they still constitute a significant part of wolf diet (Smith et al. 2000; Newsome et al. 2016). The non‐lethal effects of wolves on American bison seem to be limited. For example, a comparison of elk ( *Cervus canadensis* ) and American bison habitat use in areas with and without wolf presence in Yellowstone National Park (YNP) showed that elk avoided open areas when wolves were present, whereas no such response was found in bison (Hernández and Laundré 2005). On the other hand, a study by Laundré et al. (2001), also conducted in YNP, showed that bison increased their vigilance levels in response to wolves, specifically females. After the reintroduction of wolves in YNP, 14 American bison kills were documented between 1995 and 1999. Most of these kills occurred during the colder seasons, when bison were either in poor condition, injured, or juvenile (Smith et al. 2000).

In this context, and in contrast to observations from North American systems, the apparent rarity of European bison‐wolf interactions in the BPF and European systems in general raises questions about whether the species' classification as a non‐prey species is fully justified. Based on historical data and recent reports of predation attempts, the labelling of the European bison as a non‐prey species may be inaccurate. There appears to be a lack of knowledge on the extent of European bison‐wolf interactions, as well as a lack of contemporary, direct observations. Such observations, of both attempted and successful predation events, remain scarce. This limits our understanding of whether and how wolves may influence European bison behaviour and ecology in modern populations. Such observations are relevant as they provide insight into predator–prey interactions, anti‐predator responses, and the potential ecological role of wolves in systems where both species coexist. In this study, we present the first video‐recorded predation attempt by wolves on European bison and discuss its relevance for understanding wolf‐bison interactions.

## 2 Methods and Results

The wolf attack on European bison was recorded in the Polish part of the Białowieża Primaeval Forest (BPF). The BPF is the oldest and most well‐preserved lowland temperate forest in Europe, consisting of various habitat types ranging from dry coniferous forests to marshlands (Modzelewska et al. 2020). The BPF houses a variety of mammal species, including five ungulate species: European bison, red deer, roe deer, moose ( *Alces alces* ) and wild boar, as well as two large predators: wolf (four packs of 8–12 individuals in winter) and Eurasian lynx (approximately 15 individuals) (Schmidt and Kuijper 2015; Bubnicki et al. 2019). The European bison population on the Polish side of the forest is currently estimated to surpass 870 individuals in the BPF. Since the bison is a protected species, hunting is generally forbidden.

The video of the wolf attack on European bison was recorded on a Browning Dark Ops Pro X 1080 trail camera. The camera trap was attached to a tree and was set to make 20 s video‐recordings with a 1 s interval between videos, though during day hours the camera could record up to 2 min maximum if consistent triggering occurred. The camera trap which captured the predation attempt was part of a bigger project consisting of 57 camera traps monitoring large mammal presence throughout the year (starting in April 2025).

**FIGURE 1:**
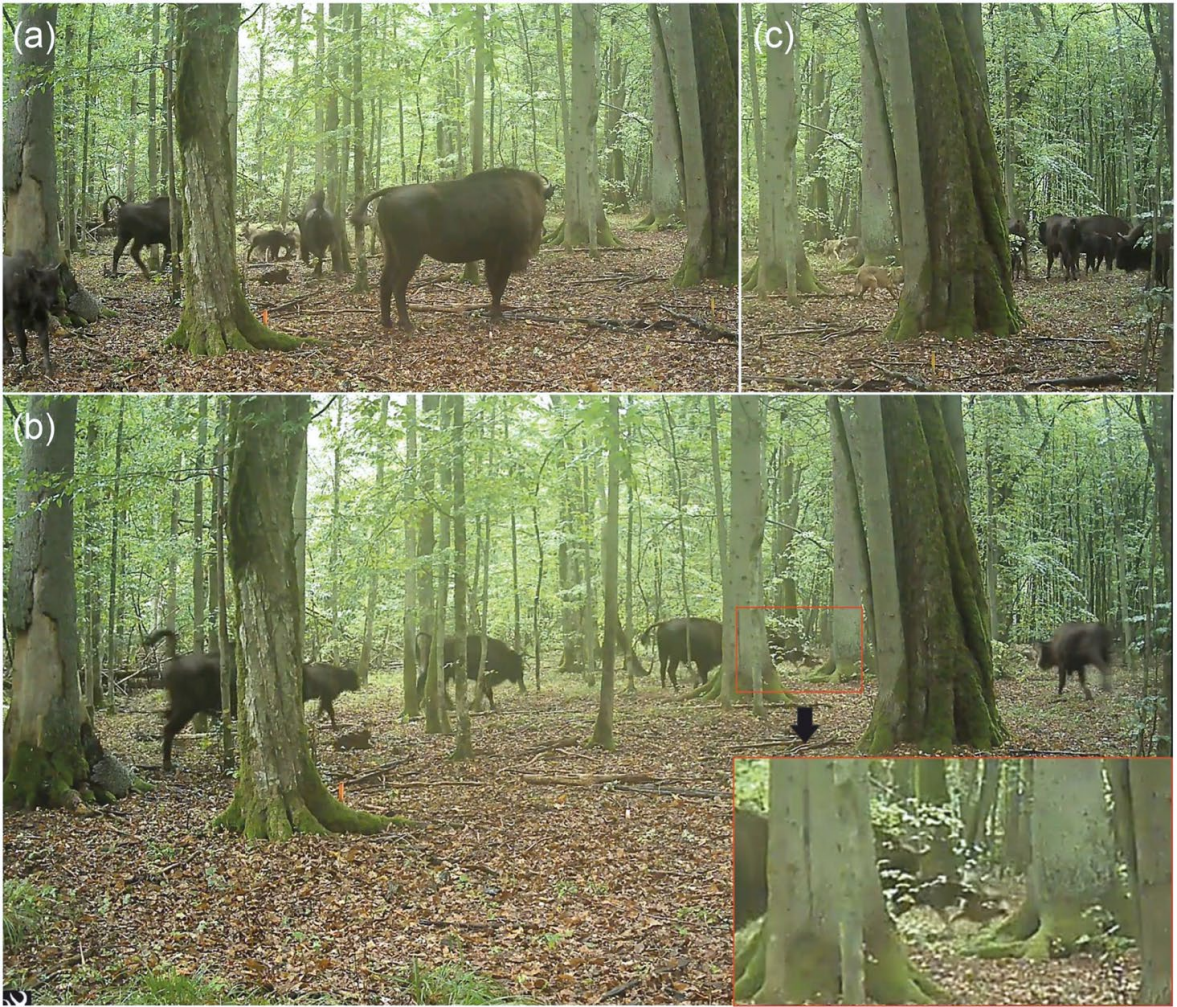
Wolves attack a herd of European bison in the Białowieża Primaeval Forest. (a) Wolves grab a newborn European bison calf while two adult cows charge towards the wolves to defend the calf. (b) Wolves grab the newborn European bison calf for the second time. The rest of the bison herd comes running in aid of the juvenile. (c) European bison circle around the newborn calf which was previously targeted twice during the wolf attack. After this event, the bison move away, and the wolf pack appears to give up. *Source:* Mammal Research Institute, Polish Academy of Sciences.

The event was recorded on the 15th of September 2025, starting at 7:25 a.m. and ending at 7:47 a.m. (Video 1). The footage showed a pack of seven wolves attacking a European bison herd (*n* = 11), consisting of five adult cows, two adult bulls, one subadult bull, and three juveniles, one of which was a newborn calf (Figure 1; Video 1). The wolves focused their attack on the smallest, newborn calf. At 7:25 a.m., the wolf attack began: five wolves ran past the camera and then return into view. Three bison cows chased the wolves, which unintentionally left the newborn calf vulnerable. The wolves attacked the calf, biting its neck and attempting to drag it away, but were driven off by two adult cows (Figure 1a). After this first attempt, the calf ran away, but the wolves managed to seize it a second time (Figure 1b). This time, two adult bison charged at the wolves, aiming for the wolves with their horns, while the rest of the herd proceeded towards the calf. The adults surrounded the newborn, and the wolves seemed to terminate their attack (Figure 1c). At 7:27 a.m. one wolf ran past the camera trap, the rest of the pack or the bison herd were not within the frame. At 7:34 a.m., an adult cow was observed chasing one of the wolves away, while a second wolf trotted in the background. At 7:40 a.m., a cow and a bull walked by the camera trap, and at 7:47 a.m. an adult bull passed, indicating the end of the recorded event.

**VIDEO 1:** A pack of wolves attacks a herd of European bison in the Białowieża Primaeval Forest. In the video recording, the wolves target a newborn calf twice to which the bison herd responds by defending the calf and charging towards the wolves. *Source:* Mammal Research Institute, Polish Academy of Sciences. Video content can be viewed at https://onlinelibrary.wiley.com/doi/10.1002/ece3.73752.

## 3 Discussion

While the European bison is generally considered a non‐prey species, we provided video‐recorded evidence of a predation attempt by wolves in the Białowieża Primaeval Forest (BPF). In our recording, wolves repeatedly targeted a bison calf, and the rest of the herd responded with anti‐predator behaviour, chasing the wolves away from the juvenile. Although the video did not show a direct kill, our observation demonstrated that the European bison is in fact a potential prey for wolves. This raises questions about why predation attempts are so rare, whether the bison can still be described as a non‐prey species, and what this could mean for conservation and management of the species.

Following the extinction and reintroduction of the European bison in the BPF, bison have been reintroduced in various parts of Europe as a part of bigger reintroduction and rewilding initiatives. Studies modelling habitat suitability for reintroductions, mainly focus on landcover (e.g., habitat type), elevation, and factors indexing anthropogenic pressure, disregarding both the non‐lethal and the lethal effects of predation risk by natural predators (Bleyhl et al. 2015; Lord et al. 2019). Predation is often mentioned as an afterthought, potentially causing mortality of a few individuals (e.g., Dănilă et al. 2022). However, there are reintroduction reports that mention predation attempts. For example, Aldea et al. (2022) reported predation attempts by brown bears and wolves in the Fagaras mountains, although no direct kills were observed. This might indicate that the European bison is a difficult prey species for large predators, a pattern also suggested by our observation, but predation attempts still occur. It is important to note that the lack of reported attempts and kills does not necessarily indicate a lack of predation events, as most such interactions are likely to go undetected.

In North America, MacNulty et al. (2014) recorded a successful attack by a wolf pack consisting of 13 individuals that captured an adult American bison, observed using a drone. The authors noted that pack size strongly impacted hunting success. According to their findings, two to six wolves are sufficient to capture an elk, whereas the optimal pack size for capturing and killing an adult American bison ranges from nine to 13 wolves. Similarly, historical reports from the BPF in the nineteenth century highlighted that attacks on European bison occurred in larger wolf packs of up to 10 individuals (Jędrzejewska and Jędrzejewski 2001). This indicates that European bison are difficult prey, especially for smaller wolf packs. In European ecosystems, the average pack size consists of 5–6 individuals, which is optimal for hunting their main prey, red deer (Jędrzejewski et al. 2004). In the BPF, the biggest wolf pack consisted of eight individuals back in 1997 (Jędrzejewski et al. 2004). At present, the maximum (winter) wolf pack size can range from five to 21 individuals (Mammal Research Institute, Polish Academy of Sciences, unpublished data; Wijnands, personal observation), indicating a clear increase in pack sizes. Wolf pack size may be a limiting factor in predating European bison, as previous numbers might not have been sufficient to hunt such large and dangerous prey. Indeed, in our observation, the wolf pack size of seven individuals may have been inadequate to successfully kill the bison. However, with wolf pack sizes expanding, the probability of bison becoming a more common prey species might increase, with potentially cascading effects. This may also occur in other parts of Europe, where wolf numbers are continuing to grow (Kuijper et al. 2016). How the increases in wolf numbers and pack size affect predation events on European bison requires further investigation.

Reports from the nineteenth century in the BPF describe that during the majority of attempted attacks, bison would not always flee, but rather relied on defensive behaviour, such as charging or directing their head and horns towards the wolves. The success or failure of a hunting attempt is reliant on both the composition and size of the predator group as well as those of the prey group. Carbyn and Trottier (1988) suggested that herds composed of individuals of both sexes and various ages provide a more effective defence against predators. This was also observed in our video‐recording, where the wolves targeted a small calf, which should provide an easy opportunity because of its small body size and vulnerability. However, the adults in the herd defended the individual by charging towards the wolves, causing them to abort their attack on the juvenile. In a video posted by “Żubry Online” on Facebook (Żubry Online 2017), an attempted wolf attack on bison was recorded during the winter of 2017. In this video, a small pack of four wolves runs towards a group of bison, which respond by chasing the wolves, after which the wolves appear to abandon the predation attempt. Similar behaviour has been observed in free‐ranging cattle. In recent years, wolves have recolonised large parts of their former range, and species that lived without predators for over a century now have to co‐exist with them. Smit and Kuijper (2024) recorded a wolf attack on free‐ranging cattle in the Netherlands, where wolves targeted a Galloway calf. The herd protected the calf by charging at the wolves, chasing them away. It is apparent that European bison are capable of defending themselves against wolf attacks, and due to their large body size, it requires a large wolf pack to take down and kill a bison, especially adults. Bison calves, however, may be more vulnerable to predation, since they are small and cannot yet defend themselves (Jung 2011). Considering that bison possess effective defence strategies, non‐lethal effects are expected to be limited. Indeed, Theuerkauf and Rouys (2008) found that bison did not exhibit behavioural adaptations as a response to wolf predation risk. This could be due to the generally low predation risk reducing the need for adaptive responses. However, if predation becomes more frequent, non‐lethal effects may emerge, potentially influencing habitat use, activity patterns, vigilance levels or physiological stress.

In this context, an important aspect of both lethal and non‐lethal effects of predation on prey is physiological stress. Stress responses can either be acute or chronic, where acute stress is triggered by a direct threat and stress levels normally decrease once the event is over (Wingfield and Kitaysky 2002). Chronic stress can be caused by persevering and unpredictable factors, with potential negative effects on fertility and body condition (Boonstra et al. 1998). Stress responses of prey to predators do not always result in chronic stress, as stress can be omitted through behavioural adaptations (Tomasulo et al. 2026). In fact, it is even expected that chronic stress is absent in prey living among predators (Boonstra 2013). Indeed, Metrione et al. (2020) demonstrated an acute stress response following a wolf attack on American bison, but found no indication of chronic stress. In the BPF, European bison may experience acute stress during wolf encounters, but due to the low and inconsistent predation risk, it is unlikely to develop into chronic stress.

### 3.1 Conservation Implications

The success of conservation efforts and minimal culling have caused European bison numbers to increase significantly. Currently, there are 10 countries in Europe hosting free‐ranging European bison, with approximately 9762 individuals estimated in 2024 (Raczyński and Bołbot 2024). Although this achievement is a huge conservation success, there are mixed responses towards the presence of the European bison. Overpopulation of bison can damage habitats, indirectly affecting other mammal species, or cause bison to seek forage elsewhere, specifically croplands during winter (Olech and Perzanowski 2014). In Knyszyn forest, the bison population increased from 68 individuals to 123 individuals in the period 2008–2014. This increase in bison resulted in enhanced damage to crops, with economic damage ranging from €12,398 to €85,921 on a yearly basis (Sobczuk and Olech 2016). While culling European bison is generally avoided due to its protected status, predation by natural predators could potentially aid in maintaining population numbers if this occurs on a larger scale (Jung 2011).

Reintroduction and rewilding initiatives aiming to restore the European bison across its former range have generally assumed that predation plays a negligible role in population establishment or conservation success. However, as wolf ranges expand and population numbers increase, their interactions with large herbivores such as European bison may change. Such interactions may influence predator–prey dynamics not only through direct mortality, but also through behavioural responses such as vigilance, grouping, habitat use, and calf protection. This should be considered when assessing the ecological functioning of rewilded systems involving large predators and European bison. If the predation on European bison occurs on a greater scale than previously assumed, it could potentially contribute to naturally slowing the population growth, thereby heading towards a reduction in human‐bison conflicts. Yet, there remains a lack of up‐to‐date knowledge on interactions between European bison and large predators, especially with the recent increase in both bison and wolf population numbers. Therefore, future studies should focus on both the lethal and non‐lethal effects of predators on European bison in order to inform bison conservation strategies.

In conclusion, although no direct kills were observed, we provided evidence that the European bison is a potential prey for wolves. In North American systems, bison are a confirmed prey species, whereas in European systems bison are often labelled as “non‐prey” due to the small number of reported kills in the past 70 years. Even though confirmed kills are rare, the previously mentioned reports and our video‐recorded observation suggest that the European bison should no longer be classified as a non‐prey species. Additionally, there is a clear lack of an overview on how often bison are targeted by predators and how frequently such attempts are successful. Our finding may have implications for the conservation and future reintroductions of European bison in areas where they co‐exist with large predators. If predation on European bison, specifically young animals, occurs more frequently than previously assumed, wolves could play a small but potentially important role in the natural regulation of bison numbers. In turn, such regulation could possibly lead to mitigation of human‐bison conflicts.

## Author Contributions

**Robin Rozemarijn Wijnands:** conceptualization (equal), writing – original draft (lead), writing – review and editing (equal). **Tomasz Borowik:** conceptualization (equal), writing – original draft (supporting), writing – review and editing (equal).

## Funding

This work was supported by Narodowe Centrum Nauki, 2023/50/O/NZ8/00152.

## Conflicts of Interest

The authors declare no conflicts of interest.

## Data Availability

No datasets were generated or analysed during the current study. All relevant data, such as video material and photos, are included in the note. Video material was edited to remove empty frames and present the event described in the note as clearly as possible.
